# Roar Data: Redefining a Lion's Roar Using Machine Learning

**DOI:** 10.1002/ece3.72474

**Published:** 2025-11-20

**Authors:** Jonathan Growcott, Alex Lobora, Andrew Markham, Charlotte E. Searle, Johan Wahlström, Matthew Wijers, Benno I. Simmons

**Affiliations:** ^1^ Centre for Ecology and Conservation, College of Life and Environmental Sciences University of Exeter Exeter UK; ^2^ Wildlife Conservation Research Unit, The Recanati‐Kaplan Centre, Department of Biology University of Oxford Oxford UK; ^3^ Tanzania Wildlife Research Institute Arusha Tanzania; ^4^ Department of Computer Science University of Oxford Oxford UK; ^5^ Lion Landscapes Iringa Tanzania; ^6^ Department of Computer Science University of Exeter Exeter UK

## Abstract

For territorial advertisement and intra‐pride communication African lions emit a roaring bout, of which one component, is their iconic roar. The full‐throated roar of a lion has recently been shown to be a unique and individually identifiable signature. At the same time, the frequency of large‐scale passive acoustic monitoring surveys has increased. As such, a lion's roar may soon become a useful tool to count individuals and estimate population density, to supplement traditional survey techniques. Currently, selecting full‐throated roars is heavily dependent on expert inference and is therefore subject to human‐induced bias. We propose a data‐driven approach to automatically classify lions' full‐throated roars from the other vocalisations that constitute a roaring bout. By using two‐state Gaussian Hidden‐Markov Models, we also demonstrate that two types of roars exist within a lion's roaring bout—a full‐throated roar and a newly named intermediary roar—and these can be classified at an accuracy of 84.7%. We further demonstrate that using simple metrics to describe lion vocalisations—maximum frequency (Hz) and vocalisation length (s)—and *K*‐means clustering is sufficient to classify lion call types, at a high accuracy (95.4%), and that using data‐driven predicted full‐throated roars results in an improved ability to identify individuals (F1‐score 0.87 vs. manual full‐throated roar classification 0.80). Here, we establish an easy‐to‐understand and implement process that will reduce the knowledge gap and make passive acoustic monitoring more accessible in a field currently dominated by other monitoring techniques (e.g., camera surveys), paving the way for novel research.

## Graphical abstract

Lion's produce two distinct types of roars. Lion vocalisations can be classified using simple acoustic parameters. Automated classification of lion roars improves individual identification.

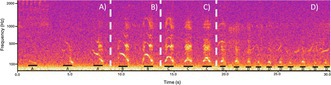

## 1 Introduction

Sound is a highly important aspect of ecology, providing information that informs interspecific and intraspecific behaviour. Acoustic communication is widespread in tetrapods but can vary in complexity (Jorgewich‐Cohen et al. 2022; Odom et al. 2021). Depending on the context, an animal may alter the sound it produces, whereby species will sometimes emit single calls, and at other times will produce different phrases or calls within a wider vocalisation (Bradbury and Vehrencamp 2011). One species that will at times produce individual vocalisations and at others emit a string of different call types is the African lion, *Panthera leo* .

The lion's roar is one of the most iconic and recognisable sounds produced by the animal kingdom. However, the roar is also only one of the many vocalisations that lions emit. Lions broadcast sound through a variety of call types including mews, snarls, chuffs, grunts, moans and roars (Rudnai 1973; Peters 2011; Stander and Stander 1988; McComb et al. 1994). Some calls are specific to ontogenetic growth, for example, mews (Peters 2011); others are closely related to specific behavioural events such as mating or fighting and as such are produced in isolation, for example, snarls (Rudnai 1973), whilst some are emitted more regularly, for example, roars (Wijers et al. 2018).

Most previous research conducted on lion vocalisations has focused exclusively on the roar (reviewed in Wijers 2019). The roar is the primary form of long‐distance communication for lions because it is of the greatest amplitude and is used to maintain contact with distant pride members or to advertise territory ownership (McComb et al. 1994; Grinnell and McComb 2001). Therefore, an individual's roar is assumed to contain information that a lion can use to assess unknown conspecifics: sex, age, number of individuals (Pfefferle et al. 2007), as well as knowledge about known individuals such as identity (Wijers et al. 2020). Both males and females are known to roar (McComb et al. 1993, 1994); however, beyond this, the frequency of roaring can be impacted by social and environmental factors. For instance, roaring is less frequently observed in individuals younger than 2.5 years (Vanherle 2011) and nomadic males, who lack prides and an established territory, will remain silent irrespective of spatiotemporal boundaries, and at the expense of maintaining social ties with other males in their coalition (Grinnell and McComb 2001). Furthermore, lions vocalise most often in the hours before dawn (Wijers et al. 2021; Schaller 1972; Stander and Stander 1988) and will also choose to roar in areas close to water (Wijers et al. 2021). As such, for lions, the act of roaring is a complex, adaptive behaviour that is influenced by the risks and rewards associated with advertising their location (McComb et al. 1993, 1994; Grinnell and McComb 2001).

Although rare, when lions roar, researchers and practitioners can also use this advertisement of location as a monitoring tool (Mwampeta et al. 2024). In 1974, Rodgers hypothesised that lion roars could be used to deduce the minimum number of lions in Selous Game Reserve, Tanzania. However, when compared to a reference population count, roaring estimates were significantly lower. Therefore, index‐based roar counts may underestimate the size of lion populations (Bauer and Van Der Merwe 2004). Whilst roar counts may have value in estimating occupancy, currently, they are not recommended for lion abundance estimation (Mwampeta et al. 2024). However, because a lion's full‐throated roar is unique to the individual (Wijers et al. 2020), more robust statistical population methods such as acoustic, spatially explicit capture–recapture models, which are widely used to monitor populations of large carnivores (Searle et al. 2025; Sollmann et al. 2013; Farhadinia et al. 2021) may offer a more promising alternative to roar counts (Efford et al. 2009; Stevenson et al. 2015). Additionally, passive acoustic monitoring surveys may be advantageous when compared to other methods used to obtain population density data such as spoor surveys or camera traps because of their larger detection ranges (Crunchant et al. 2020). As such, a lion's roar may yet be a useful tool for estimating population sizes.

**FIGURE 1:**
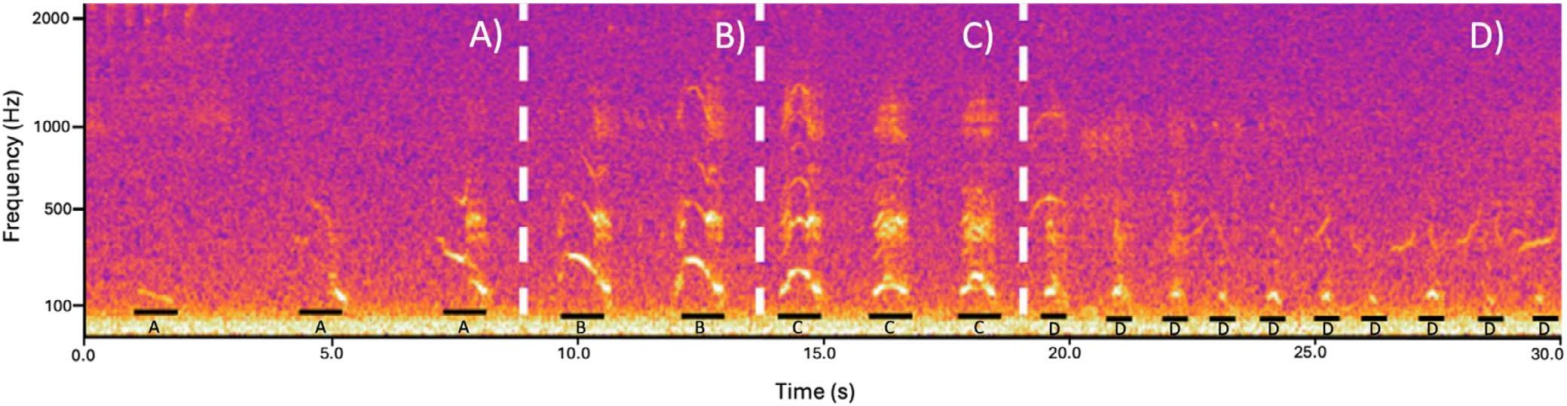
A spectrogram of a lion's roaring bout. Outlined, A–D, are the four stages. A constitutes moans, B constitutes full‐throated roars, C constitutes what we propose to be intermediary roars and D constitutes grunts. Ordinarily, intermediary roars are exclusively found after the full‐throated roars and before the grunts. An example of a lion's roaring bout can be found in Audio 1.

If lion roars are to be used for population monitoring, then it is imperative that there is a clear and consistent understanding as to what constitutes a full‐throated roar to prevent human‐induced classification errors from researchers and practitioners. Previously, vocalisations within a lion's roaring bout have been categorised into three distinct call types: moans, full‐throated roars and grunts (Stander and Stander 1988; McComb et al. 1994). Each section is distinguishable by sound and spectrogram signature. However, during a roaring bout, initial full‐throated roars diminish in duration and maximum frequency, and the temporal pattern alters (Figure 1). Indeed, in the seminal study demonstrating that lions have unique, individually identifiable roars, Wijers et al. (2020) only used the first three full‐throated roar units from each roaring bout. However, as there are high levels of intra‐individual and inter‐individual variation in the number of roars emitted per roaring bout (Stander and Stander 1988; Schaller 1972; Wijers et al. 2020) there is reason to believe that the classification of full‐throated roars should come from the acoustic features intrinsic to the vocalisation and not an arbitrary heuristic.

**AUDIO 1:** An example of a lion's roaring bout. Audio content can be viewed at https://onlinelibrary.wiley.com/doi/10.1002/ece3.72474.

First, we propose that within a lion's roaring bout there are four distinct call types: moan, full‐throated roar, intermediary roar (which has not previously been classified), and grunt and that these vocalisations within each class can be grouped together using Hidden‐Markov models. Second, using *K*‐means clustering, we suggest that intermediary roars can be differentiated from full‐throated roars solely by their reduced duration and maximum frequency. Third, we anticipate that a *K*‐means clustering, data‐driven approach to classifying full‐throated roars will result in an improved ability to discriminate between individuals within a population.

## 2 Methods

### 2.1 Investigating Four Distinct Call Types Within a Lion's Roaring Bout

#### 2.1.1 Study Site

**FIGURE 2:**
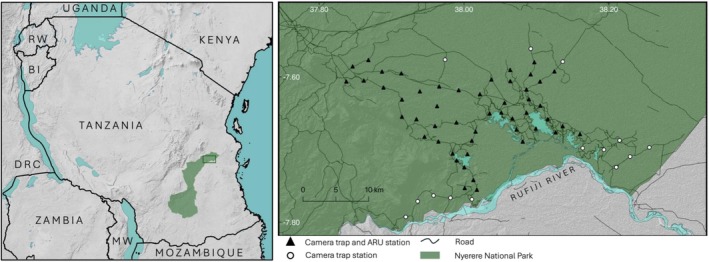
Left: The location of Nyerere National Park within Tanzania (World Terrain Base by Esri, NASA, WDPA). Right: Detail of the paired camera trap and ARU deployment in the Matambwe sector of Nyerere NP, comprising a total of 50 ARU and camera trap stations and 14 camera trap‐only stations.

The study took place in the Matambwe sector of Nyerere National Park, which lies in southern Tanzania and between the latitudes of −7.505° and −7.823° and longitudes of 37.728° and 38.308° (Figure 2). Vegetation is dominated by floodplain grassland and *Acacia* woodland (Creel and Creel 2002). There are two distinct seasons in southern Tanzania: a dry season from May to October and a wet season from November until April, with an average rainfall of ~1400 mm (Creel and Creel 2002). Permission to conduct research in Nyerere National Park was granted by the Tanzania Wildlife Research Institute (TAWIRI), Tanzania National Parks Authority (TANAPA) and the Commission for Science and Technology (COSTECH), under research permits 2023‐780‐NA‐2023‐879 and 2023‐665‐ER‐2021‐287.

#### 2.1.2 Data Collection

We deployed paired xenon white‐flash camera traps (Cuddeback Professional Colour Model 1347; Non Typical Inc., WI, USA), at a total of 64 stations (average spacing 2.54 km) along roads and trails in the study area. To acquire directly comparable audio data, at 50 of these stations, we also installed CARACALs, which are custom‐built, low‐cost autonomous recording units (ARUs) that record in quadrophonic format via four active microphones (M1:M4) positioned on the printed circuit board at 90° intervals (Wijers et al. 2019). From herein, we will only focus on the ARU component of the survey, but full methods can be found in Growcott et al. (2024).

The entire ARU deployment was active for 62 days between 20 September and 20 November 2023. CARACALs were installed in trees at heights between 2 and 4 m. Care was taken to ensure the M1 microphone was positioned facing northward and that the devices were parallel to the ground. Batteries and micro‐SD cards were changed approximately every 21 days. CARACALs recorded audio data continuously at a 16 kHz sampling rate and 32 bits per sample; data were saved in 1‐h files in .wav format.

#### 2.1.3 Extraction of Roaring Bouts

A random sample of audio data was manually inspected in Audacity 3.4.2 (Audacity Team 2024) for lion roaring bouts. One‐hour files were first given a binary classification: lion roars detected or not. Positive files were then visually inspected in Raven Pro (Version 1.6.5) and processed manually. Lions often roar in chorus (McComb et al. 1994); therefore, only roaring bouts that did not contain overlap were annotated to ensure vocalisations could be reliably assumed to be from a single individual. Additionally, we did not include incomplete roaring bouts. Incomplete bouts are those that do not contain all expected vocalisations (moans, roars, grunts) either because the lion did not produce them or, more commonly, because not all vocalisations were recorded. The individual calls within solo, complete roaring bouts were annotated by JG with tightly fitting bounding boxes (Figure S1). Using the bounding boxes and Raven's in‐built selection tables, two simple metrics that describe the spectrogram features of the calls were exported: maximum frequency (Hz) and Delta Time (seconds). Raven's in‐built annotation selection extraction process was then used to export individual calls in 16 kHz sampling rate and 32‐bits per sample .wav format. Call type dataset sizes were balanced; whereby, a random selection of calls was selected from each call type that met the lowest class size.

Using R version 4.4.4 (R Core Team 2022), the fundamental frequency contour (the F0 contour), which is the lowest frequency of a periodic waveform (Figure 1), for each individual call was automatically extracted. First, each call was subjected to a digital Butterworth bandpass filter between the frequencies of 30 and 375 Hz, using a 2048‐point moving Hann window with 68% overlap. The extracted F0 was computed using a window size of 128 samples and by selecting the top 1% of the signal's amplitude. The extraction process was conducted using the autoc() function from the *seewave* package (Sueur et al. 2008).

### 2.2 Hidden‐Markov Model Classification of Lion Call Types

A Hidden‐Markov Model (HMM) allows probability distributions to be modelled over a sequence of observations. As such, we used two‐state Gaussian HMMs to model the temporal pattern of each call type found with a lion's roaring bout using their F0 contour. Here, the two states reflect the hidden path (in two parts) for the observed sequence of the F0 contour. Each individual call was treated independently. Each call was used once as the test sample whilst the remaining calls formed the training data to build an HMM for each call type. The test call was then compared with each HMM using log‐likelihood to determine which HMM the test call was most likely to be assigned to. The HMM that produced the highest log‐likelihood value was determined to be the predicted class. For each unique test call, the HMMs were rebuilt using all the data with that call excluded. The entire process was repeated over 1000 iterations with randomised parameter initialisations. HMM classification was performed in Python version 3.9.13 using the ‘hmmlearn’ library (Hmmlearn Development Team 2024). The HMM classification was recorded in a confusion matrix, number of True Positives (TP), False Positives (FP), True Negatives (TN) and False Negatives (FN). Performance was then assessed using four metrics that were calculated as follows: accuracy ((TP + TN)/(TP + TN + FP + FN)), recall (TP/(TP + FN)), precision (TP/(TP + FP)) and F1‐score (2(precision × recall)/(precision + recall)). We report these metrics for each call type and the overall classification performance.

### 2.3 *K*‐Means Clustering of Lion Roaring Bout Call Types

To test whether different calls produced within a lion's roaring bout can be automatically classified, we used *K*‐means clustering (MacQueen 1967)—an unsupervised machine learning algorithm that classifies data to a predetermined number of clusters—to predict moans, full‐throated roars, intermediary roars and grunts based on maximum frequency and duration of the vocalisation. For each variable, data were standardised (mean‐centred and divided by the standard deviation). *K*‐means clustering was performed with the STATS package in R (R Core Team 2022).

#### 2.3.1 Case Study

To determine whether using a data‐driven approach to classify full‐throated roars resulted in an improved ability to discriminate between individuals within a population, we tested our classification approach on data collected from Wijers et al. (2020).

##### 2.3.1.1 Study Site

The study took place in Bubye Valley Conservancy (BVC), a privately owned wildlife area located in southern Zimbabwe between latitudes −21.209° and −21.851° and longitudes of 29.789° and 30.521° East measuring approximately 3400 km^2^ in area.

##### 2.3.1.2 Data Collection

###### 2.3.1.2.1 Animal‐Borne Acoustic‐Accelerometer Biologgers

In November 2014, custom‐designed biologgers were fitted to five male and three female lions in BVC (Wijers et al. 2018). Each device comprised a triaxial accelerometer and magnetometer sampling at 32 Hz per axis and a mono‐electret microphone sampling audio at 16 Hz with 8‐bit resolution (frequency response ~30 Hz–8 kHz; dynamic range ~40 dB). All components were encased in an epoxy resin reinforced housing with a hydrophobic vent provided for the microphone. Study animals were chemically immobilised using 75–100 mg Zoletil (Virbac RSA (Pty) Ltd., Halfway House, South Africa) combined with 5 mg medetomidine (Kyron Laboratories, Johannesburg, South Africa). Immobilisation drugs were delivered intramuscularly by 1 cc darts (Pneudart, Williamsport, PA, USA) projected from a Dan‐Inject CO_2_‐ pressurised dartgun (Dan‐Inject, Børkop, Denmark). After fitting the biologger, ~25 mg atipamazol (Antisedan, Pfizer Animal Health, Johannesburg, South Africa) was administered to reverse the effects of medetomidine allowing the animal to recover within 15–90 min. The biologgers recorded continuous audio (8‐bit, 16 kHz mono) for between 4 and 10 days before the batteries were depleted. Study animals were then recaptured and the data downloaded for processing and analysis.

###### 2.3.1.2.2 Labelling of Vocalisations

Again, all lion audio recordings were processed manually in Raven Pro (Version 1.6.5) by visually inspecting spectrograms and drawing tight bounding boxes around the fundamental frequency of lion vocalisations (Figure S1). To ensure that lion vocalisations were taken exclusively from a single individual, only lion roaring bouts which contained zero overlap were used. Labelling of full‐throated roars occurred in two different ways. First, labels were manually proscribed to each call type. Second, *K*‐means clustering, as previously described, was used to predict full‐throated roars. The cluster deemed to represent full‐throated roars was selected through graphical observation.

We repeated our methodology of using HMMs, as previously described, to model the temporal pattern of the F0 contour for each individual lion. However, here, to prevent bias resulting from temporal autocorrelation between roars within the same bout, we used leave‐one‐out cross‐validation, whereby the test bout was kept separate from bouts included within the training data. Every bout was used once as a test sample and the remaining bouts formed the training data to build an HMM for each lion.

## 3 Results

### 3.1 Lion Roaring Bouts

From our Nyerere National Park, Tanzania dataset, lion vocalisations were extracted from 27 of the 350 randomly selected files from nine stations (out of 25 sampled stations). By chance, there was no temporal overlap between files from different stations, but this did prevent pseudo‐replication of vocalisations. From these, 1416 vocalisations were manually classified: 197 moans, 211 full‐throated roars, 195 intermediary roars and 813 grunts. Comparatively, 1733 vocalisations were extracted from the five male lions collared at BVC: 115 moans, 199 full‐throated roars, 99 intermediary roars and 1320 grunts. Unexpectedly, none of the female lions emitted roaring bouts during the study; however, this was attributed to the presence of small cubs in the pride (Wijers et al. 2020).

### 3.2 HMM Classification of Lion Call Types

**FIGURE 3:**
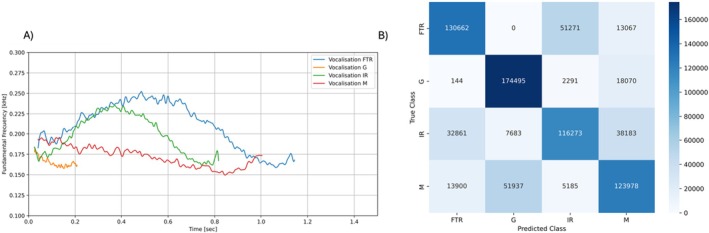
(A) Average fundamental frequency contours for every vocalisation type. Acronyms represent each call type: FTR, full‐throated roar; G, grunt; IR, intermediary roar; M, moan. (B) Confusion matrix of true and predicted vocalisation types. Each vocalisation was assigned to the most likely call type HMM 1000 times. Model training and testing were carried out on separate vocalisations.

Call types within a lion's roaring bout differed (Figure 3A). Modelling the temporal pattern of different F0 call type sequences, using 195 vocalisations for each call type, resulted in good classification performance with a micro: 84.7% accuracy, 70.9% recall, 68.8% precision and 70.0% F1‐score. Performance varied across individual call types (Figure 3B). The lowest and highest F1‐scores were intermediary roars (62.8%) and grunts (81.3%).

### 3.3 *K*‐Means Clustering of Lion Call Types

**FIGURE 4:**
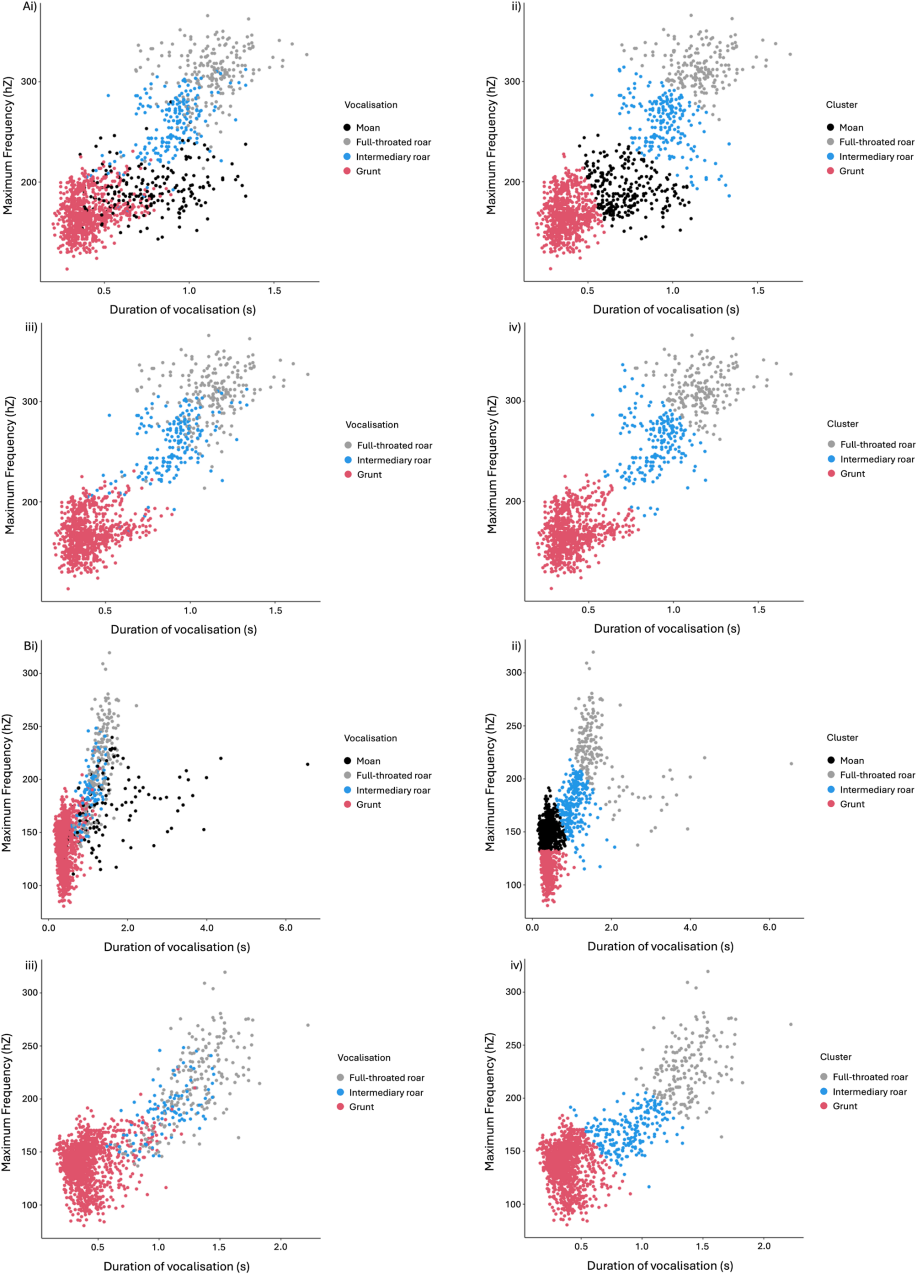
Comparison of true labels (left) and predicted labels (right) of lion vocalisations found within a roaring bout with duration of vocalisation (s) and maximum frequency (Hz) as input features, using the *K*‐means clustering algorithm. (A) Data presented were extracted from Nyerere National Park, Tanzania. (B) Data presented were extracted from Bubye Valley Conservancy, Zimbabwe (Wijers et al. 2020). Each study site's predictions are shown firstly (i, ii) with all vocalisation types and secondly, with moans manually removed (iii, iv). Note the different horizontal axis for A and B.

Attempting to cluster into four classes, one cluster for each call type, exhibited varying success (Figure 4). Classification performance was high and consistently improved when moans were excluded; thus, using three clusters to predict (Table 1). Lower BVC precision and recall may be attributed to uncertain initial human classification because in Wijers et al. (2020), only three roars were classified as full‐throated roars per bout. Indeed, low classification of intermediary roars was seen, with 33.3% of intermediary roars predicted to be full‐throated roars (Figure 4Biii,iv). In comparison, Nyerere National Park intermediary roars were only classified as full‐throated roars at an error rate of 11.3% (Figure 4Aiii,iv).

**TABLE 1:** *K*‐means clustering performance metrics for each study site, with and without moans.

Study site	Accuracy (%)	Precision (%)	Recall (%)	F1‐score
Nyerere National Park (with moans)	91.3	76.3	77.9	76.7
Nyerere National Park (without moans)	95.4	87.8	87.1	87
Bubye Valley Conservancy (with moans)	68.6	47.9	43.7	38.5
Bubye Valley Conservancy (without moans)	92.1	69.9	76.5	70.8

### 3.4 Individual Identification of Zimbabwean Lions

**FIGURE 5:**
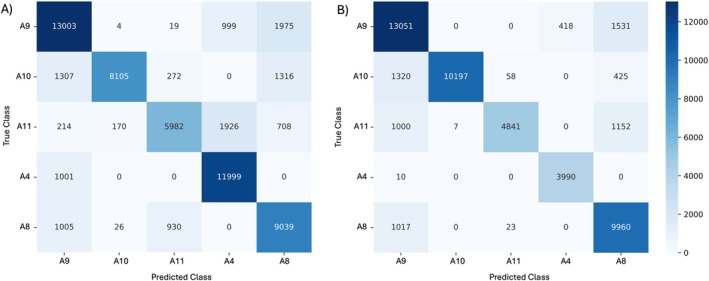
Confusion matrices of true and predicted lions for lion full‐throated roars. Each lion bout was assigned to the most likely lion HMM 1000 times. Model training and testing were carried out on separate bouts. Full‐throated roars were classified manually (A) and automatically (B).

Classification of individual lions varied depending on the methodology used to select full‐throated roars (Figure 5). When full‐throated roars were selected manually high levels of classification were achieved with a macro: 92.1% accuracy, 79.2% recall, 81.8% precision and 0.80 F1‐score. However, classification performance was improved further when full‐throated roars were selected using *K*‐means clustering with a macro: 94.3% accuracy, 86.3% recall, 88.9% precision and 0.87 F1‐score. Performance varied across individual lions (Table 2), although individuals' F1 scores ranked in the same order based on both manual and automatic selection. An important difference between the two methods used was the number of full‐throated roars used as training data: a total of 155 full‐throated roars were manually extracted (mean [±SE] 31.0 ± 7.80 per lion), whereas 179 were automatically predicted (mean [±SE] 35.8 ± 9.90 per lion). With the exception of one individual (A4, for whom five full‐throated roars were predicted), *K*‐means clustering predicted more vocalisations to be classified as full‐throated roars than manual classification for each individual (Table 3).

**TABLE 2:** Model performance metrics for each individual lion. Ordered by automatic F1‐score.

Lion	Accuracy (%)		Precision (%)		Recall (%)		F1‐score	
	Manual	Automatic	Manual	Automatic	Manual	Automatic	Manual	Automatic
A4	93.5	99.1	80.4	90.5	92.3	99.8	0.86	0.95
A10	94.8	96.3	97.6	99.9	73.7	85.0	0.84	0.92
A9	89.1	89.2	78.7	79.6	81.3	87.0	0.80	0.83
A8	90.1	91.5	69.3	76.2	82.2	90.5	0.75	0.83
A11	92.9	95.4	83.0	98.4	66.5	69.2	0.74	0.81

**TABLE 3:** Comparison of the number of bouts and total number of roars for each individual lion across classification methods.

Individual	A9	A10	A11	A4	A8
Sex (M/F)	M	M	M	M	M
Number of bouts					
Manual	16	12	9	13	11
Automatic	15	12	7	4	11
Total number of roars					
Manual	61	26	18	30	20
Automatic	66	43	29	5	36

## 4 Discussion

This study presents a human‐in‐the‐loop classification system to differentiate vocalisations within a lion's roaring bout. We show for the first time that lions produce four call types within a roaring bout, including two distinctive types of roars. We provide evidence that using a posteriori understanding of a species' vocalisations can successfully reduce the number of spectrogram features required to automatically classify call types, thus avoiding more complex methodology. We consolidate the importance of our human‐in‐the‐loop classification system by demonstrating that a data‐driven approach to classify full‐throated roars, which contain an individual's identity, improves our ability to identify unique lions.

Before now, only one form of lion roar was believed to exist within a roaring bout (Stander and Stander 1988; McComb et al. 1994). Here, we suggest that lion roars should instead be separated into full‐throated roars and intermediary roars. Our finding that lion vocalisations are more complex than previously thought is in keeping with other large carnivores that produce iconic vocalisations. Initially, spotted hyaenas ( *Crocuta crocuta* ) were thought to produce three forms of whoop within a bout (East and Hofer 1991); however, more recently, this understanding has been refined and altered to suggest that there are four forms of whoop—preliminary, symmetric, asymmetric and terminal—as described in Lehmann et al. (2022). Preliminary and asymmetric whoops appear similar when viewed on a spectrogram; however, preliminary whoops only occur at the start of a bout (Lehmann et al. 2022). Moreover, asymmetric whoops are more unique to an individual than preliminary whoops. Therefore, asymmetric whoops are a better vocalisation to use when differentiating individuals by vocalisation (Lehmann et al. 2022). Including this temporal element when differentiating vocalisations has been important as it now allows for different researchers to be consistent when labelling vocalisation types and to improve the performance of downstream analysis. But still, similar to spotted hyaenas, further research is required to determine if lions are conveying specific information within the individual sub‐sections of their roaring bouts, and whether intermediary roars carry a different behavioural and evolutionary purpose compared to full‐throated roars.

### 4.1 Data‐Driven Classification

By introducing a data‐driven approach to classify lion vocalisations, we establish a reproducible, transferable framework for lion bio‐acousticians. Annotating and labelling acoustic datasets can be heavily dependent on human perception (Nguyen Hong Duc et al. 2021; van Osta et al. 2023). Thus, an automated classification process helps to remove some level of reliance on human inferred subjectivity; however, other aspects will remain, for example, identifying the start and end time of a vocalisation. Another benefit of data‐driven classification is that audio analysis can become more accessible as an analytical tool for non‐expert users. Using bioacoustics and associated analyses to monitor cetaceans or birds is commonplace and has been used for many decades (Gibb et al. 2019), but for large carnivore research, large‐scale acoustic studies have only recently begun (Growcott et al. 2024). Reviews such as Stowell (2022) and Kershenbaum et al. (2025) provide clear instructions for how best to use computational techniques for bioacoustics generally, but species‐specific protocols are just as important when attempting to increase participation in developing research fields and as demonstrated in this study, can also help optimise performance.

Whilst we provide a relatively straight forward approach to automatically differentiate between three call types found within a lion's roaring bout, we appreciate that our methodology does still rely on an element of human subjectivity by having to initially discard moans and as such is a human‐in‐the‐loop rather than a fully automated process. However, we believe that manually classifying moans does not result in an increased workload or require expert knowledge. Moans can easily be identified by their sound, spectrogram signature, and place within a roaring bout. Lions will initialise their roaring bouts with moans, and these will only occur before the first full‐throated roar has taken place (Stander and Stander 1988; McComb et al. 1994). Whilst moans can have high variability in maximum frequency and duration—hence their unsuitability to being clustered using solely maximum frequency and duration as predictors—their spectrogram signature does not arch in the same way that a full‐throated roar does (Figures 1 and 3A). As such, manual classification of moans can be carried out easily alongside the extraction of all other vocalisations. With future research it may be possible to fully automate the process; however, this would not reduce time spent extracting calls. Moreover, given the relative importance of full‐throated roars compared to other vocalisations within a roaring bout, the priority and major motivation of this study was to discriminate between the different roars rather than full automation.

### 4.2 Simplicity vs. Complexity

In comparison to other recently published studies that have attempted to automatically differentiate between different vocalisation types from the same species, our methodology is deliberately simple. A popular technique has been to use predefined acoustical features, such as temporal and spectral features as well as intensity of the signal, followed by dimensionality feature reduction to select the optimal predictors (Elie and Theunissen 2016; Best et al. 2023; Clink and Klinck 2021; Sainburg et al. 2020). We avoided this step by initially visually inspecting our targeted vocalisations, which led to a clear understanding of the specific spectrogram features that could be targeted to facilitate accurate clustering. Similarly, more complex and more computationally taxing auto‐encoder embeddings were avoided. Auto‐encoders are artificial neural networks trained using self‐supervised learning to encode data into a lower dimensional space (Best et al. 2023). Auto‐encoders trained on vocalisation spectrograms have shown promising results (Goffinet et al. 2021; Bergler et al. 2019; Best et al. 2023) and may facilitate full automation of lion vocalisations within a roaring bout. However, optimal performance appears to be driven by training data sizes. Best et al. (2023) found that Normalised Mutual Information (NMI) scores between auto‐encoder predictions and expert labels were highest for species that had datasets consisting of at least 140,000 vocalisations. For species such as lions, where data collection is much harder because they exist across large home ranges (Zehnder et al. 2018), or vocalise sporadically (Wijers et al. 2020), deep learning methods, that rely upon custom architecture and substantial training data, may not be optimal. However, this may soon alter; the emergence of foundational models in the bioacoustics field to perform few‐shot learning using a well‐trained encoder on a proxy task may be of particular use for analysing the vocalisations of cryptic, vocally elusive species (van Merriënboer et al. 2024). Nevertheless, importantly, in our instance, the need for high‐level computational infrastructure was not necessary because high levels of classification were obtained regardless.

### 4.3 Geographical Variation in Lion Roars—Lion Accents?

Whilst we have demonstrated that full‐throated roars can be identified at the population level, only five of the 30 human‐classified full‐throated roars for A4 lion were identified when clustering. This low performance may be attributed to two factors: (1) A4 did not produce full‐throated roars within his roaring bouts; (2) classification was impacted by A4's full‐throated roars having a lower maximum frequency or shorter duration than the other lions (Wijers et al. 2020). The second factor could be explained by A4's origin: unlike the other lions in the Zimbabwean study, A4 was known to have originated from the Tuli Block in eastern Botswana, more than 60 km from Bubye Valley Conservancy, Zimbabwe. Indeed, Wijers et al. (2020) alluded to lions from different geographic origins emitting discernible roars. Similarly, Stander and Stander (1988) observed that lions from Etosha National Park, Namibia, had shorter roars compared with lions from other parts of Africa. Likewise, within this study, the maximum frequency and duration of full‐throated roars produced by lions in southern Tanzania and western Zimbabwe differed (Figure 4). Differences in maximum frequency and duration of full‐throated roars between populations of lions may notionally impact the application of this study. Nearly 20% of lion populations in open, unmanaged systems consist of nomadic males who can disperse > 200 km from their natal pride (Funston et al. 2003). If the full‐throated roars of these individuals are shorter, or at a lower maximum frequency than the rest of the population there is a risk that these individuals would be filtered out. Further research that identifies the extent to which geographic variation may impact the acoustic parameters of full‐throated roars would help address these concerns.

## 5 Conclusions

Using bioacoustics to study large African carnivores is an emerging direction of research (Wijers 2019; Lehmann et al. 2022; Growcott et al. 2024). However, because species such as lions, leopards ( *Panthera pardus* ), spotted hyaenas and African wild dogs ( *Lycaon pictus* ) all produce loud, long‐distance individually identifiable vocalisations (Wijers et al. 2020; Growcott et al. 2024; Lehmann et al. 2022; Hartwig 1995), technology like passive acoustic monitoring could exploit such traits to act as a useful monitoring tool. Establishing easy‐to‐understand processes, such as those we describe here, that show lions have more than one type of roar and that these can easily be clustered apart to reduce human annotation bias and improve individual identification, will make passive acoustic monitoring more accessible in a field primarily dominated by other monitoring techniques (Mwampeta 2023). Yet, because bioacoustics is still in its infancy, there needs to be a paradigm shift for large‐scale uptake. If passive acoustic monitoring for large African carnivores is to properly develop, large‐scale and long‐term understanding of its advantages and disadvantages in relation to other monitoring techniques needs to be rigorously assessed, while ensuring that recommendations from reviews such as Kershenbaum et al. (2025), which advocate for interdisciplinary collaborations that maximise cross‐discipline expertise, are implemented. Regardless, here, we demonstrate that by implementing technology and machine learning in new scenarios, it provides us with the opportunity to analyse in greater detail and re‐evaluate ecological phenomena as famous as, a lion's roar.

## Author Contributions

**Jonathan Growcott:** conceptualization (lead), data curation (lead), formal analysis (lead), funding acquisition (lead), investigation (lead), methodology (lead), project administration (lead), writing – original draft (lead), writing – review and editing (equal). **Alex Lobora:** project administration (supporting), writing – review and editing (equal). **Andrew Markham:** resources (equal), software (equal), writing – review and editing (equal). **Charlotte E. Searle:** funding acquisition (lead), project administration (equal), resources (equal), writing – review and editing (equal). **Johan Wahlström:** supervision (equal), writing – review and editing (equal). **Matthew Wijers:** conceptualization (equal), data curation (equal), supervision (equal), writing – review and editing (equal). **Benno I. Simmons:** conceptualization (supporting), methodology (supporting), project administration (equal), supervision (equal), writing – review and editing (equal).

## Disclosure

Statement on Inclusion: Our study brings together authors from a number of different countries, including the country in which this study was based. Authors were engaged early on and before any fieldwork commenced. Whenever possible, our research was discussed with local ecologists to build capacity for conservation technology in the landscape.

## Conflicts of Interest

The authors declare no conflicts of interest.

## Supporting information


**Figure S1:** ece372474‐sup‐0001‐FigureS1.docx.

## Data Availability

All the data required are available in the Supporting Information.
