# Supplementary material for: Roar Data: Redefining a Lion's Roar Using Machine Learning
Source: Ecol Evol. 2025 Nov 20;15(11):e72474. doi: 10.1002/ece3.72474 (PMC12998244; doi:10.1002/ece3.72474)
Supplement: Supplementary file 2 — Figure S1: ece372474‐sup‐0001‐FigureS1.docx. [file ECE3-15-e72474-s001.docx]

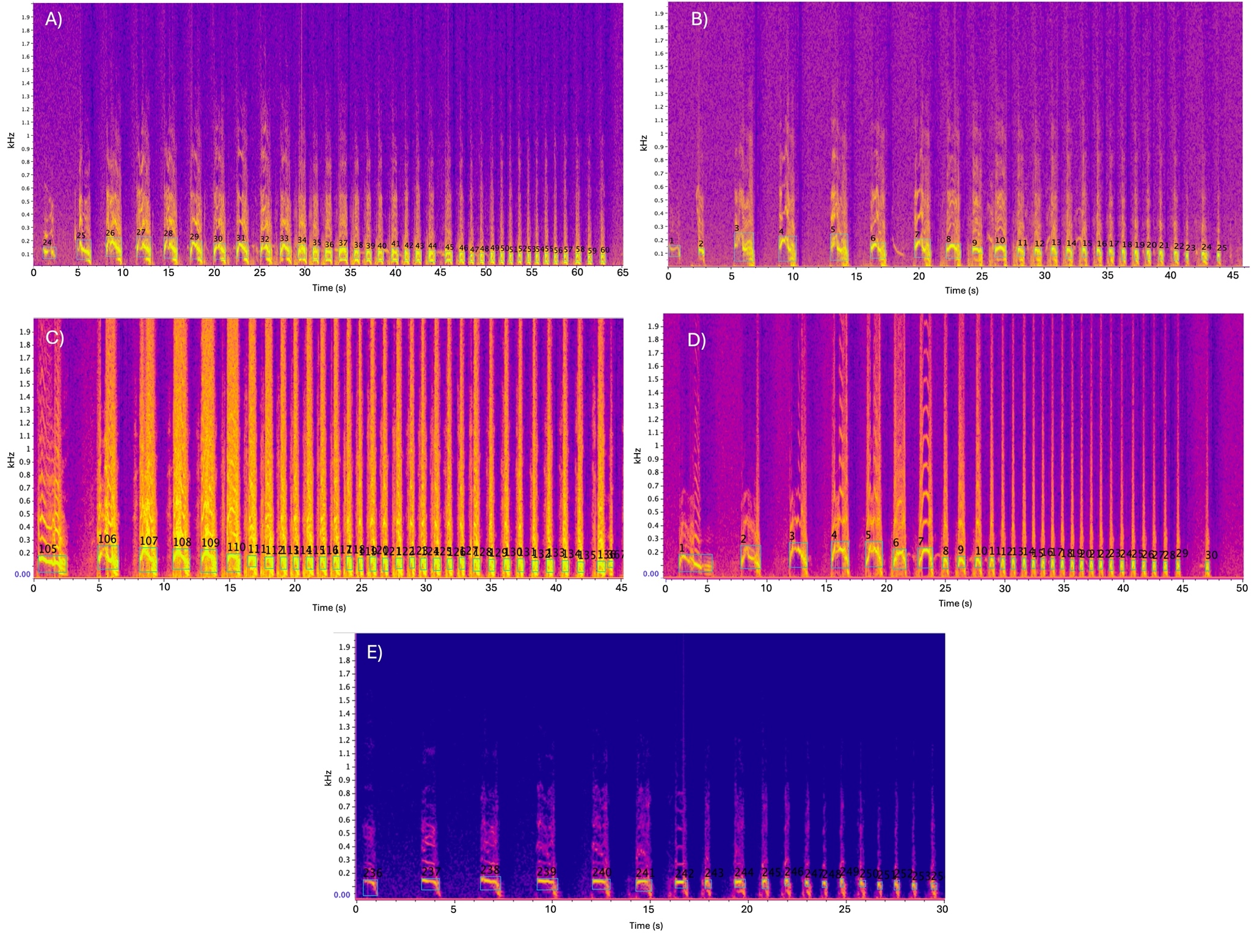
Figure S1. Examples of lion roaring bouts, with bounding boxes for each vocalisation, from each of the five lions collared in Zimbabwe. In order: A) A9 B) A10 C) A11 D) A8 E) A4.

Audio S1. An example of a lion’s roaring bout.
